# Supporting primary care clinicians in caring for patients with alcohol use disorder: study protocol for Records for Alcohol Care Enhancement (RACE), a factorial four-arm randomized trial

**DOI:** 10.1186/s13722-024-00526-x

**Published:** 2025-02-05

**Authors:** Kara M. Magane, Richard Saitz, Sarah Fielman, Marc R. LaRochelle, Christopher W. Shanahan, Christine A. Pace, Michael LaValley, Kaley Penington, Skylar Karzhevsky, Emily Hurstak

**Affiliations:** 1 Department of Community Health Sciences, Boston University School of Public Health, 801 Massachusetts Ave, Boston, MA 02118 USA; 2 Section of General Internal Medicine, Department of Medicine, Boston University Chobanian & Avedisian School of Medicine and Boston Medical Center, 801 Massachusetts Avenue, Crosstown 2, Boston, MA 02118 USA; 3 Clinical Addiction Research and Education Unit, Boston University School of Medicine and Boston Medical Center, 801 Massachusetts Ave, Boston, MA 02118 USA; 4 Department of Biostatistics, Boston University School of Public Health, 801 Massachusetts Ave, Boston, MA 02118 USA; 5 Talent Groups, 800 Town & Country Blvd, Suite 500, Houston, TX 77024 USA; 6 Department of Health Law, Policy & Management, Boston University School of Public Health, 715 Albany St, Boston, MA 02118 USA

**Keywords:** Alcoholism, Alcohol-related disorders, Electronic health records, Primary care

## Abstract

**Background:**

Unhealthy alcohol use, a spectrum of use inclusive of risky consumption and alcohol use disorder (AUD), is a leading cause of preventable death in the United States. Most people with unhealthy alcohol use do not receive evidence-based treatment. This four-arm factorial design randomized trial will assess whether population health management (PHM) and clinical care management (CCM) support for primary care providers (PCPs) are associated with improved AUD treatment engagement among their patients, beyond electronic health record (EHR) prompting and decision support alone.

**Methods:**

PCPs from an urban safety-net hospital-based primary care clinic are randomized to one of four groups (1) EHR best practice advisory (BPA) and clinical decision support tools for unhealthy alcohol use (BPA), (2) BPA plus population health manager support, (3) BPA plus clinical care manager support, and (4) all three. All PCPs will have access to the EHR BPA and decision support tools which provide chart-based advisories and order set navigation. PCPs assigned to receive PHM support will receive quarterly panel-level feedback on AUD treatment metrics for their patients. PCPs assigned to receive CCM support will receive CCM facilitation of AUD treatment processes including medication counseling, referrals, and support through direct patient interactions. The primary outcome will be the percent of patients engaged in AUD treatment among those with a new AUD diagnosis on a PCP’s panel. Secondary outcomes include the percent of patients with a new diagnosis of AUD who (1) initiated AUD treatment, (2) were prescribed AUD medications within 90 days, and (3) numerical counts of a range of AUD health services (outpatient encounters, specialty AUD care encounters, referrals, and acute healthcare utilization) in this sample. We will assess the primary outcome and the acute healthcare utilization secondary outcomes using Medicaid claims; the remaining secondary outcomes will be assessed using EHR data.

**Discussion:**

The study will evaluate how a targeted EHR innovation alone, compared with population health and care management enhancements alone or in combination, impact engagement in AUD treatment, a national quality of care measure. Findings will advance understanding of supports needed to improve systems of care for AUD in general settings.

**Trial registration:**

ClinicalTrials.gov identifier/registration number (NCT number): NCT05492942

**Supplementary Information:**

The online version contains supplementary material available at 10.1186/s13722-024-00526-x.

## Background

Unhealthy alcohol use, a spectrum of alcohol use including risky consumption through alcohol use disorder (AUD), is highly prevalent and associated with increased morbidity and mortality [1–7], particularly among patient groups at risk for health disparities [8, 9]. Despite a high frequency of contact with the healthcare system, most people with unhealthy alcohol use do not receive evidence-based interventions to reduce harm [10]. This is likely due to a variety of factors such as stigma, the perception of alcohol as less harmful than other substances, challenges with implementing and maintaining alcohol screening in general medical settings, and time or prioritization constraints in busy clinical environments [11–13]. Given these challenges, electronic health records (EHRs), used in most primary care practices, have great potential to enhance care related to unhealthy alcohol use, particularly when paired with clinical decision support (CDS) and used in conjunction with population health management (PHM) or clinical care management (CCM).

EHR innovations, including embedded decisional tools and best practice advisories, have demonstrated improved outcomes for screening and management of chronic conditions [14–17]. Several integrated health systems have used EHRs (e.g., Veterans Health Administration, Kaiser Permanente, etc.) to improve healthcare delivery for chronic conditions in primary care settings [18–22]; however, these interventions have been supported by significant system-specific infrastructure. EHRs have been shown to improve the management of chronic diseases (e.g., diabetes [23]) through Best Practice Advisories (BPAs) – reminder tools within the EHR providing clinician decision support, creation of registries to aggregate information about the target population, and by assisting the clinician in disease-specific care management through an electronic order SmartSet [24, 25]. However, EHRs alone may not surmount the barriers to increasing patient identification, delivering brief interventions, and increasing referrals for AUD treatment [18, 26]. In addition, as EHR alerts proliferate, busy and overwhelmed providers may experience alert fatigue and ignore them [27, 28]. When paired with targeted staff support, such as a population health manager and clinical care manager, EHRs may better assist clinicians in identifying, assessing, treating, and monitoring care for chronic medical conditions [29–33].

Population health management involves efforts to improve the identification and management of health conditions for a clinical population with a shared medical condition through activities such as creating registries to improve identification of the condition, classification of the status of the condition (i.e. severity), performance on quality metrics including associated outcomes or complications, and identification of care gaps [25, 29, 30]. Support from a population health manager equipped with a registry to track outcomes and treatments was associated with improved completion of laboratory testing and lower hospitalization rates for patients with diabetes [34]. PHM has been used to improve outcomes for multiple chronic health conditions such as asthma, diabetes in persons living with HIV, and chronic kidney disease, and to increase preventative health screening [35–39]. PHM is increasingly utilized in primary care settings to improve chronic disease health outcomes through targeted clinical outreach for specific health conditions and to provide assistance around social determinants of health factors [40]. When well-designed, PHM alerts that use electronic health record techonology have the potential to decrease clinical burnout [41].

Clinical care management, in contrast, is a patient-facing intervention designed to assist clinicians, patients, and their support systems in managing medical conditions. It has been historically focused on complex, high-cost patients or medical conditions [42, 43]. Clinical care managers working with patients with AUD can improve alcohol-related care by educating patients, building motivation for change, and removing patient-level and provider-level barriers to facilitate referrals to AUD treatment and medication initiation [44]. Previous studies have demonstrated that CCM embedded in primary care increases engagement in care [45] and reduces heavy drinking [31]. CCM facilitates and coordinates alcohol-related care that otherwise may not be prioritized and follows patients longitudinally to determine the outcomes of those care processes. CCM can also enhance the longitudinal relationship many primary care providers (PCPs) have with their patients by providing another trusted team member to help patients achieve their goals. CCM, envisioned as complex care management in some primary care settings, focuses on patients with medical complexity or complexity related to social determinants of health needs (lack of housing, low health literacy, etc.) to lower emergency room and hospitalization rates and improve health outcomes with variable success [46, 47].

In summary, the identification of AUD and receipt of evidence-based treatments for AUD is poor in general health settings. Although the reasons and solutions are many and complex, EHRs have great potential to improve AUD care at a low cost. Enhanced identification of AUD can be achieved with registries, which collate existing data in EHRs and then can prompt members of the clinical team to the possibility of unhealthy alcohol use or AUD using BPAs. Decision support can increase the provision of evidence-based AUD care through simplified order sets and targeted education tools deployed during a visit. However, in an era of EHR alert proliferation and increasing demands on PCPs during and after the patient encounter, adding enhancements to EHR BPA tools, such as PHM and CCM support, may further increase receipt of evidence-based AUD care by providing clinicians with additional team members to assist in the identification of patients and facilitation of AUD treatment services. The potential benefits of these supports may be particularly pronounced in clinical practices that serve populations in underserved communities where the prevalence of AUD is high, and patients may experience disproportionate barriers to receiving evidence-based care [48]. This study will evaluate these three clinician support systems alone and in combination on AUD outcomes among their primary care patients in a large, urban, safety-net hospital-based primary care clinic with a diverse patient population at risk for health disparities.

## Methods/design

### Study design

The **R**ecords for **A**lcohol **C**are **E**nhancement (RACE) study is a four-arm randomized trial that will test feasibility and obtain preliminary effectiveness estimates comparing (1) clinician prompting via an EHR-based Best Practice Advisory (BPA) and CDS alone (hereafter this intervention is referred to as “BPA”), (2) BPA plus population health management (BPA + PHM), (3) BPA plus clinical care management (BPA + CCM) and (4) all three (BPA + PHM + CCM), on AUD treatment engagement and other patient outcomes. The RACE study follows a two-by-two factorial trial design for the PHM and CCM interventions, with all randomized clinicians also receiving the BPA condition. PCPs are the unit of randomization and recipients of the intervention. The intervention period will last 18 months; however some clinicians may receive less than 18 months of the intervention depending upon when they enroll in the trial and in the event of an unplanned or unanticipated departure from the clinic (see Table 1 for schedule of enrollment, interventions, and measures). The trial outcomes will be assessed in primary care patients with AUD empaneled to PCPs enrolled and randomized.

**Table 1:** Schedule of enrollment, interventions, and measures

	Study Period^a^			
	Enrollment	Allocation	Intervention	Post Intervention
PCP Recruitment (emails and in-person)	X			
PCP Informed Consent^b^	X			
PCP Baseline Survey^b^	X			
Randomization		X		
EHR Supports (BPA)	X^c^	X^c^	X	X^c^
PHM compiles and sends quarterly reports, and prompts front desk scheduling for patients on weekly report			X	
CCM generates and reviews weekly reports, facilitates patient AUD care and provides direct patient interaction			X	
CCM and PHM complete weekly fidelity to the intervention assessments			X	
Study Team collects Medicaid Claims Data to Assess Trial Outcomes^b^			X	X
Study team collects EHR data to assess trial outcomes^b^			X	X
PCP Follow-up Survey^b^				X

PCP = Primary Care Provider; EHR = Electronic Health Record; BPA = Best Practice Advisory; PHM = Population Health Manager; CCM = Clinical Care Manager; AUD = Alcohol Use Disorder. ^a^Adverse events (AEs) and other unintended effects of trial interventions or trial conduct will not be collected systematically during the study period. The collection approach for AEs during the study period will be non-systematic assessment (i.e. AEs will be collected in response to spontaneous reports by participants). The study team (including the Principal Investigators) will assess any spontaneously reported AEs, and AEs will be reported to the Institutional Review Board. No concomitant care provided outside of the scope of the trial will be prohibited during the study period; routine clinical care will continue to be available to all patient participants throughout the duration of the trial. ^b^Any personal information and identifiers collected during the study will be stored securely. As an additional protection, this study is covered by a Certificate of Confidentiality. ^c^ Electronic health record supports were designed and implemented during the preparatory phase of this project preceding the start of trial enrollment. These electronic health record supports for unhealthy alcohol use and alcohol use disorder are anticipated to remain in use by the clinic as part of standard clinic processes and electronic health record tools available to PCPs

This primary outcome (AUD treatment engagement following a new AUD diagnosis episode) is based on the National Committee for Quality Assurance (NCQA) Healthcare Effectiveness Data and Information Set (HEDIS) quality of care measure for initiation and engagement of substance use disorder treatment (IET) [49]. A new AUD diagnosis is defined as a healthcare service in which a patient receives an AUD diagnosis when there has not been an AUD diagnosis for a healthcare service during the prior 194 days, excluding diagnoses assigned in the emergency department or detoxification settings. NCQA defines these encounters as new AUD diagnosis episodes, which are eligible for treatment initiation and engagement [49]. Initiation of treatment following a new diagnosis episode is defined as receiving a healthcare service or medication for AUD within 14 days of the new diagnosis, and engagement is defined as receiving two or more additional healthcare services or AUD medication within 34 days of initiating treatment.

We hypothesize that compared to the BPA alone, BPA combined with PHM and CCM separately (BPA + PHM vs. BPA and BPA + CCM vs. BPA) and all three together (BPA + PHM + CCM vs. BPA), will improve rates of AUD treatment engagement following a new AUD diagnosis, and other AUD care outcomes. To account for multiple testing, the significance level for the three hypotheses will be adjusted by the Bonferroni correction to 0.0167.

### Study setting

The study is being conducted in the adult general internal medicine (GIM) primary care clinic based within an urban, academic, safety-net hospital system serving a patient population that is approximately 30% Black, 60% White, and 10% other races, with 25% of individuals identifying as Hispanic. The practice has approximately 150 clinicians, including attending physicians, nurse practitioners, and resident clinicians who deliver care through approximately 130,000 visits annually.

### Study participants, recruitment, and randomization

Primary care clinicians (attending physicians, physicians in fellowship training, resident physicians, and nurse practitioners) who care for adult primary care patients in the GIM clinic and who are expected to maintain their current position in the practice for a minimum of 18 months were recruited via email (information sent to clinicians with a link to a website with additional study details) and in-person in the GIM clinic. Clinicians interested in enrolling provided written informed consent electronically via a REDCap [50, 51] e-Consent process approved by the Boston University Medical Campus Institutional Review Board. Clinician participants were enrolled from November 2022-July 2023. After clinicians were enrolled, a statistician generated the allocation sequence using SAS statistical programming software. Randomization was stratified by clinician type (e.g., nurse practitioner, attending physician, resident, or fellow physician) and by the estimated number of patients with a new AUD diagnosis assigned to the clinician’s panel based on recent historical data for their panel (stratification was based on whether the clinician was above or below the average in patients with a new AUD diagnosis for all clinicians in the practice). Allocation was concealed from study staff until the moment of intervention assignment. After assignment, clinicians are informed of their intervention assignment via email from the study team. Due to the nature of the interventions, blinding clinician participants to their assigned allocation was not possible. Throughout the intervention period, patient records (healthcare claims data and electronic health record data) that contribute to the primary and secondary outcomes are collected and will be utilized to assess trial outcomes. Patient eligibility criteria for record collection are: assigned to a randomized clinician’s primary care panel, age ≥ 18 years, *≥* 1 completed encounter in the GIM clinic in the prior 18 months, and eligible for alcohol-related care based on high-risk alcohol screening results (described below) or an alcohol-related clinical (International Classification of Disease, ICD) diagnosis. A waiver of informed consent was obtained from the Boston University Medical Campus Institutional Review Board for patient records in the trial.

### Study conditions/interventions

#### Electronic health record Best Practice Advisory (BPA) and Clinical Decision Support (CDS)

Epic Systems, the largest EHR vendor, is the electronic health record software utilized by the health system within which the study primary care clinic resides, with the capacity to design and implement various CDS tools. For the RACE study intervention, the study team (inclusive of primary care and addiction medicine clinicians) updated and refined the health system’s existing Epic-based unhealthy alcohol use and AUD BPAs and clinical decision supports. The BPA instantaneously alerts the clinician of their patient’s potential needs regarding evaluation and management of unhealthy alcohol use. The BPA activates (becomes visible to a clinician in the patient’s chart) during point of care in the event of recent (past 30 days) positive alcohol screening results (i.e. single-question alcohol screening [52] which asks “how many times in the past year have you had X drinks in a day, where X is 5 for men 65 years of age or younger, and 4 for women and men over 65 years of age; a response of *≥* 1 day is positive) and/or Alcohol Use Disorder Identification Test [AUDIT] [53] score consistent with risky alcohol use (2 to 13/15 [female/male]) or AUDIT score suggestive of AUD (*≥* 13/15 [female/male]). The BPA also activates when a patient has a 100% alcohol-attributable diagnosis [54] in the health record (active on their problem list or clinical encounter diagnoses) documented in the prior 30 days. The BPA does not create a “hard stop”; in other words, the provider does not have to acknowledge it in order to continue charting. It provides the clinician with concise, actionable information including patients’ alcohol screening results and an interpretation of those screening results, with a linked clickable option to record a new AUDIT screening result and/or evaluate the patient with the Diagnostic and Statistical Manual of Mental Disorders (DSM) criteria for AUD. The BPA links to CDS directly to facilitate easy opening of this tool by clinicians. The CDS is an Epic SmartSet based on best practice guidelines for the management of unhealthy alcohol use and AUD. The SmartSet provides decision support to facilitate diagnosing AUD, providing brief intervention for unhealthy alcohol use, ordering labs relevant to the clinical management of AUD, prescribing AUD pharmacotherapy, placing referrals for behavioral health services (psychologist, psychiatrist, social worker, etc.), placing referrals for AUD specialty care (office based addiction treatment clinic, young adult addiction treatment clinic, etc.), and for accessing and printing patient educational materials regarding unhealthy alcohol use and AUD. See Additional file 1 for images and further details of the BPA and CDS.

#### BPA + PHM

All clinicians randomized to the BPA + PHM arm will have access to the BPA and CDS EHR tools as detailed above. Additionally, they will receive support from a population health manager (PHM). The PHM is an existing Population Health Manager embedded in the GIM clinic with time devoted to improving care for chronic medical conditions, who was provided with dedicated and protected time and effort on the RACE study (approximately 4 h per week on average during the study intervention period) to fulfill the study PHM tasks and responsibilities during the study intervention period. The PHM will access and run monthly registry-based “workbench reports” in Epic to examine clinician panel-level AUD quality metrics (detailed in Table 2) for clinicians randomized to PHM. Reporting “workbench” is an Epic tool that can produce customized reports that display rows of data, and can be sorted and filtered by end users. In support of these registry-based workbench reports, the study team developed a clinically useful, live (continuously updated with real-time inputs based on health record data) Alcohol Registry in Epic of patients who have screened positive on alcohol screening and/or have a diagnosis suggestive of AUD (i.e. a 100% alcohol-attributable diagnosis [54]) on their problem list in the EHR. The Alcohol Registry includes data from EHR fields including but not limited to alcohol screening results, alcohol-related diagnoses, AUD pharmacotherapy prescribed, and referrals for alcohol counseling/behavioral health and office-based based addiction treatment.

**Table 2:** Population Health Management reports for the Records for Alcohol Care Enhancement (RACE) study intervention

Clinical Care Domain	Report Name	Denominator	Numerator
Alcohol Screening	AUDIT Receipt	Patients on the PCP’s panel who had a positive single-question alcohol screening result in the past calendar month	Patients who completed the AUDIT on the same day as their positive result on the single-question alcohol screening
	AUDIT result suggesting harmful use	Patients on the PCP’s panel who received the AUDIT in the past calendar month	Patients who had a positive AUDIT score suggestive of harmful or hazardous alcohol use (score of *≥* 8)
	AUDIT result suggesting AUD	Patients on the PCP’s panel who received the AUDIT during the past calendar month	Patients who had a positive AUDIT score suggestive of AUD (score of *≥* 13/15 [male/female] or *≥* 4 on the AUDIT dependence questions)
	Alcohol-related diagnosis in the presence of positive AUDIT result	Patients on the PCP’s panel with an AUDIT score *≥* 13/15 or *≥* 4 on AUDIT dependence questions during the past calendar month	Patients with a 100% alcohol attributable diagnosis on their EHR problem list
AUD Care HEDIS-based Quality Metrics	Treatment Initiation	Patients on a PCP’s panel with a new^a^ AUD diagnosis received in the past month	Patients who received a healthcare service for an AUD diagnosis, or were prescribed AUD pharmacotherapy within 14 days of the new AUD diagnosis
	Treatment engagement	Patients on a PCP’s panel with new^a^ AUD diagnosis who have initiated treatment in the past month	Patients who receive two additional AUD healthcare services (including visits or medication) within 34 days of initiating treatment
Ongoing Treatment Engagement and Utilization	AUD Pharmacotherapy	Patients on a PCP’s panel with AUD on their EHR problem list who had a visit in GIM the past month	Patients with an active prescription for AUD medication
	Specialty AUD Care	Patients on a PCP’s panel with AUD on their EHR problem list who had a visit in GIM in the past month	Patients receiving AUD specialty care in the OBAT clinic (at least one visit in the OBAT clinic in the month preceding or following the patient’s GIM encounter)

AUDIT = Alcohol Use Disorder Identification Test; PCP = Primary Care Provider; AUD = Alcohol Use Disorder; EHR = electronic health record; GIM = General Internal Medicine; OBAT = Office Based Addiction Treatment; HEDIS = Healthcare Effectiveness Data and Information Set. ^a^ New AUD diagnosis is defined as a healthcare service in which a patient receives an AUD diagnosis when there has not been an AUD diagnosis for a healthcare service during the prior 194 days excluding diagnoses assigned in the emergency department or detoxification settings

The monthly registry-based workbench reports accessed by the PHM display rows of data (typically patients) with columns displaying different variables such as the date of patient’s most recent alcohol screening, etc. The reports yield lists of patients meeting specified inclusion criteria, which are then compiled by the PHM into quarterly, aggregate summaries of each PCP’s panel-level performance on AUD quality metrics. The PHM distributes these quarterly personalized quality reports via email to clinicians. The quarterly report includes quality metrics such as the number and percent of patients on the clinician’s panel who had: positive alcohol screening results, initiated treatment within 14 days of receiving a new AUD diagnosis, and engaged in treatment within 34 days of initiating treatment. The complete list of monthly quality metric reports run by the PHM is detailed in Table 2 with definitions of how the proportion is defined (numerator/denominator). Additionally, the PHM runs a weekly workbench report in the EHR (Epic) to identify patients who had one of the following encounter types for an AUD or alcohol-related diagnosis in the prior week within the health system: emergency department, inpatient admission, or outpatient “bridge” clinic visit. For patients identified on this weekly list, the PHM sends an Epic message to GIM clinic scheduling staff, copying the patient’s PCP, requesting that a scheduler contact the patient to schedule a follow-up visit in primary care within the next two weeks to provide ongoing care related to their alcohol-related medical condition.

#### BPA + CCM

All clinicians randomized to the BPA + CCM arm will have access to the BPA and CDS EHR tools as detailed above. Additionally, they will receive support from a clinical care manager (CCM). The CCM is a registered nurse with additional training in addiction medicine. The CCM is a dedicated, part-time (averaging approximately 10 h per week) RACE study staff member embedded in the GIM clinic with access to the electronic health record and clinic information technology. The CCM accesses and runs weekly registry-based workbench reports in Epic that identify patients with an AUD diagnosis who are assigned to PCPs in the CCM arm. Specifically, workbench reports identify (1) patients who received a new AUD diagnosis in the prior seven days but have not yet initiated treatment, (2) patients with a new AUD diagnosis who initiated AUD treatment in the prior seven days but have not yet engaged in AUD treatment, (3) patients who had an encounter in the prior seven days with an AUD diagnosis (both new and established) and (4) patients who had a positive AUDIT screening result in the prior seven days but did not receive an alcohol-related diagnosis. Patient records appearing on the workbench reports can be acted upon directly by the end-user; for example from the workbench report the CCM can directly enter the patient’s chart, send the patient a MyChart message, or place orders for selected patients. The CCM regularly reviews the chart of each patient included on the workbench reports to determine which patients may need follow-up AUD care such as referrals, AUD medications, or assistance with AUD care navigation. The CCM conducts telephone outreach to these patients and communicates with clinicians to discuss potential patient care plans, and assists in implementing these care plans through the preparation of prescriptions and referrals for co-signature, direct patient counseling, and facilitation of services external to the healthcare system.

#### BPA + PHM + CCM

Clinicians randomized to the BPA + PHM + CCM arm will have access to the BPA and CDS EHR tools as detailed above. Additionally, they will receive support from the population health manager and the clinical care manager as detailed above.

### Data sources and study outcome measures

#### Data sources

##### Administrative Data

The data source for the primary outcome and select secondary outcomes will be statewide Medicaid data including inpatient encounter claims, outpatient medical and behavioral health claims, emergency department encounter claims, detox, pharmacy claims, associated diagnoses (ICD-10 codes), procedure codes, dates of service, revenue codes, and provider type. We will also use subscriber-level data including coverage enrollment dates.

##### Fidelity to the intervention assessments

To capture fidelity to the intervention, the PHM and CCM will complete a weekly checklist of intervention components, specific to their role. PHM and CCM enter fidelity to intervention data directly into REDCap. As part of the checklist review, the PHM and CCM will be asked to indicate whether various components of their intervention were completed for the prior week, including whether each workbench report was run, how many patients appeared on the reports, number of communications they had with clinicians, and (for the CCM) number of outreaches/interactions they had with patients. The study team monitors completion and responses on the fidelity checklists on a regular basis.

##### PCP surveys

Upon enrolling in the study, clinician participants are asked to complete a brief online survey in REDCap at baseline to collect clinician sociodemographic characteristics (age, gender, etc.) as well as information about their clinic role and credentials, number of years in clinical practice, addiction medicine training, and confidence managing patients with AUD. A follow-up survey is distributed to clinician participants at the end of their time in the study to reassess their addiction medicine training, and confidence managing patients with AUD, as well as an open-ended question soliciting a free-text response asking for any additional information or feedback on their experience as a participant in the trial. Other relevant clinician characteristics (e.g., title, highest degree completed) will be collected via public sources.

#### Study outcome measures

##### Primary outcome

The primary outcome is engagement in AUD treatment, based on the HEDIS national quality of care measure from the NCQA [49]. Engagement is defined as having two or more healthcare services (inclusive of AUD medication) with a diagnosis of AUD within 34 days of initiating treatment [55, 56]; initiation is defined as having a healthcare service (inclusive of medication) with a diagnosis of AUD within 14 days of a new AUD diagnosis episode [56]. A new AUD diagnosis episode is defined as a healthcare service in which a patient receives an AUD diagnosis when there has not been an AUD diagnosis for a healthcare service (excluding diagnoses assigned in the emergency department or detoxification setting) during the prior 194 days [56]. Engagement in AUD treatment is a national quality of care measure and is feasible to measure with generalizable relevance to different settings [57, 58]. Treatment engagement is associated with a reduction in mortality for individuals with a substance use disorder, lower addiction severity, especially for outpatients with AUD, improved employment and wages for individuals involved in the justice system, and fewer arrests for crimes [57, 59, 60]. Using AUD treatment engagement as the primary outcome balances what is achievable by the intervention with the potential to demonstrate improvement on a measure with significant clinical meaning and reimbursement implications for healthcare systems.

##### Secondary outcomes

Initiation (as defined above) is a secondary outcome that will be assessed using Medicaid claims. It is the most proximal measure of activity that could directly result from the intervention. Other secondary outcomes to be assessed using Medicaid claims include acute healthcare utilization (emergency department visits and hospitalizations) within 90 days of a new AUD diagnosis and acute alcohol-related healthcare utilization (emergency department visits and hospitalizations associated with a 100% alcohol-attributable diagnosis within 90 days of a new AUD diagnosis). Secondary outcomes assessed via the health system-level electronic health record data include: the proportion of patients who have been prescribed AUD medication within 90 days of a new AUD diagnosis, the number of outpatient visits with an AUD diagnosis within 90 days of a new AUD diagnosis, number of mental health clinician visits with an AUD diagnosis within 90 days of a new AUD diagnosis, number of visits for AUD specialty care within 90 days of a new AUD diagnosis, and number of referrals for counseling or specialty AUD care within 90 days of a new AUD diagnosis.

### Statistical analysis

Each of the three primary pairwise comparisons will be conducted at an alpha level of 0.0167 to maintain an overall type I error rate of 5%. Power calculations to detect the primary outcome of interest (engagement) assume 2-sided tests with an overall significance level of 0.0167. Calculations for engagement are based on a chi-square test and estimates adjusted for clustering based on the design effect with an expected interclass correlation coefficient of 0.10. With an expected 32 clinicians in each randomized group and an anticipated average of eight new AUD diagnosis episodes per clinician, we expect a sample size of patient records (i.e., patient episodes of a new AUD diagnosis during the intervention period) of approximately 1,000. Recent historical EHR data for the GIM clinic was used to estimate the anticipated average of eight new AUD diagnoses per clinician during an 18-month period. Prior data available from the larger health system consortium (of which the study’s clinic is a member), estimated the rate of treatment engagement for all substance use disorders to be 20.6% [61]. However, results from another prospective study that occurred in the primary care clinic that is the site for the current study showed that for patients with AUD diagnoses, 2–5% received specialty referrals, 1–3% received medication or detoxification services for AUD, and 2–3% were referred to Alcoholics Anonymous [62]. Based on these historical data, we estimate that 15% of new AUD diagnosis episodes will result in treatment engagement in the BPA-only condition. Therefore, the proposed study has 80% power to detect an absolute difference of 17% (i.e., 15% in the BPA only group vs. 32% in any of the three combined intervention arms) in the proportion of patients meeting criteria for treatment engagement.

We will use an intent-to-treat analysis including all eligible patients of the primary care clinician participants according to the clinician’s randomized assignment. Only patients who have had continuous Medicaid enrollment during the eligibility period for the outcome (14-day treatment initiation window and 34-day treatment engagement window, 48 days in total following the new AUD diagnosis episode) will be eligible for inclusion in the primary outcome (AUD treatment engagement) analysis. Descriptive statistics will be calculated for patient-specific and clinician-specific characteristics at baseline and used to determine any differences between randomized arms. With the unit of observation occurring at the patient level, the main analysis evaluating the effect of the interventions on the binary study outcomes will use generalized estimating equations logistic regression models with empirical standard errors to account for clustering by clinicians. Secondary confirmatory analyses will be conducted using mixed effects logistic regression models accounting for clustering by including a random effect for clinician. An additional analysis will be further adjusted for the time each clinician spent receiving the intervention in the study, accounting for late study entry and early withdrawal to see if this impacts the treatment estimates. Indicator variables will be included to represent the study arms, adjust for the randomization stratification factors including clinician type and clinician volume, and explore geographic (clinicians provide care over five geographic suites) effects not already accounted for. Models will control for baseline characteristics between groups. Spearman correlation coefficients will be obtained to identify pairs of variables that may be collinear (*r* > 0.4) and would therefore not be included together in regression analyses. In addition, the variance inflation factor will be assessed to detect possible collinearity.

## Discussion

AUD is an under-diagnosed and under-treated medical condition despite enormous personal, clinical, and economic costs both directly and indirectly related to it. While the reasons and solutions to this are many and complex, the combination of EHR tools and clinician supports employed in this study may overcome some barriers to providing evidence-based AUD care and improving receipt of high-quality care. The PHM intervention is designed to help clinicians identify the prevalence of unhealthy alcohol use among their patient panel and to know how they are performing regarding managing care for their patients with AUD, knowledge which may prompt or motivate clinicians to improve the quality of the AUD care they are providing [25, 63]. PHM does not directly assist clinicians with implementing care through direct patient contact. The CCM intervention facilitates individual patient care via direct contact between the CCM and patients to assist with implementing AUD treatment plans.

This study will operationalize a national quality of care metric as the primary and one of the secondary outcomes. The Initiation and Engagement of Substance Use Disorder Treatment (IET) measure is a performance measure in the Healthcare Effectiveness and Information Set (HEDIS) of the National Committee for Quality Assurance (NCQA). More than 227 million people are enrolled in health plans that report HEDIS results [64], and improving quality performance is a priority for many healthcare systems as a result. For the RACE study, we will use claims data to identify healthcare services meeting the criteria for alcohol-specific IET among primary care patients of trial-enrolled PCPs. Using claims data will allow us to identify AUD healthcare services and pharmacotherapy provided both within and outside of the system in which the trial is conducted. However, we can only assess many of our secondary outcomes using the health system’s EHR data, which increases the potential of underestimating certain AUD services received by patients. In addition, patients are required to have a period of continuous Medicaid enrollment during the 48-day IET window following a new AUD diagnosis episode to be eligible for inclusion in the analysis of the treatment engagement outcome. Given that coverage lapses are common in the clinic’s patient population and are known to be common among patients with substance use disorder [65, 66], this may lead to the exclusion of patients with a new AUD diagnosis for whom we are not able to assess subsequent treatment engagement due to a coverage lapse. These limitations are shared with health services research studies that use claims based data and/or electronic health record data as primary sources. We expect these limitations in our data would bias us towards a null effect or no observed effect of the interventions on improving AUD care in primary care patients at the study site.

An additional possible limitation of our protocol is that, patients are attributed to a study condition based on the PCP to whom they are assigned in the EHR, which may not always be accurate. While the primary care clinic makes efforts to maintain the accuracy and currency of this PCP assignment field in the patient chart, there are circumstances where a patient changes PCP and an update to this field is not made due to clinic staff error or inadequate patient communication about a change in where they receive primary care. Additionally, this trial will evaluate AUD outcomes for patients assigned to each randomized PCP, even though patients may have received their new AUD diagnosis and follow-up AUD care from clinicians other than their assigned PCP. The Administrative Medicaid claims data and EHR data sources that are used in this trial will contain encounter-level information including the clinician who submitted an AUD diagnosis and the clinician who provided AUD care at each AUD treatment encounter. Therefore, while it is expected that AUD diagnoses and follow-up care will be provided by clinicians other than the patient’s PCP, the study will have the requisite data available to evaluate and describe the frequency at which this occurs. Moreover, we do not consider these to be significant limitations given that primary care is in a transition period with a greater emphasis on team-based care and multidisciplinary clinician supports to achieve improved patient outcomes, lower costs, improve satisfaction among patients and clinicians, and improve retention of clinicians [67–69]. The CCM intervention in this trial will be applied at the level of the clinical team. For example, the CCM will include other team members such as nurses, patient navigators, and relevant specialty clinicians (hepatologist, psychiatrist) on messages supporting a patient’s plan for AUD care. Patients may receive care from other members of the clinical team or providers who are not their assigned PCP, which may limit the measurable impact of the PHM intervention [70, 71], we expect the CCM intervention to have some impact on the clinical team caring for the patient.

In this pragmatic trial, we implement interventions to support PCPs in a real-world, safety-net primary care clinical setting using a randomized trial methodology expected to distribute sources of bias equally among intervention groups. Due to the pragmatic nature of this trial implemented in a real-world setting, measuring fidelity to the intervention, as our study does, is important. Our tool for capturing fidelity utilizes self-report data, which are subject to recall and reporting bias. Short assessment intervals (weekly) for PHM and CCM completion of their fidelity to the intervention assessments were implemented to minimize recall bias, and adherence to these weekly intervals was monitored by other study staff members. While not formally and systematically captured, the study’s Principal Investigator provided periodic direct supervision of PHM and CCM activities, further monitoring their fidelity in implementing the interventions. Despite the inherent limitations of evaluations in a real-world setting, we expect that results obtained from the study will inform the feasibility and potential of leveraging EHRs in widespread use in an innovative way to improve the identification and management of unhealthy alcohol use. Results will inform how a set of targeted clinical interventions alone and in combination may lead to improved patient care outcomes, including increased and timely receipt of quality, evidence-based AUD treatment and reductions in costly alcohol-related acute healthcare utilization such as emergency department visits and inpatient stays. Such improvements, if realized, are likely to have desirable downstream impacts such as reduced alcohol-related morbidity and mortality. Importantly, results from this trial may inform changes and decisions made to practices and systems that could be widely disseminated, translated, and implemented in healthcare settings across the United States.

## Electronic supplementary material

Below is the link to the electronic supplementary material.

Supplementary Material 1

## Data Availability

No datasets were generated or analysed during the current study.

## Abbreviations

AE: Adverse event
AUD: Alcohol use disorder
BPA: Best practice advisory
CCM: Clinical care management (manager)
CDS: Clinical decision support
EHR: Electronic health record
HEDIS: Healthcare effectiveness data and information set
IET: Initiation and engagement of substance use disorder treatment
GIM: General internal medicine
NCQA: National committee for quality assurance
PHM: Population health management (manager)
PCP: Primary care provider
