# Supplementary material for: Supporting primary care clinicians in caring for patients with alcohol use disorder: study protocol for Records for Alcohol Care Enhancement (RACE), a factorial four-arm randomized trial
Source: Addict Sci Clin Pract. 2025 Feb 5;20:9. doi: 10.1186/s13722-024-00526-x (PMC11800519; doi:10.1186/s13722-024-00526-x)
Supplement: Supplementary file 1 — Supplementary Material 1 [file 13722_2024_526_MOESM1_ESM.pdf]

Targeted Users: Family Medicine, Internal Medicine, General Internal Medicine, & Addiction Medicine
Release date: 4/9/2021

Alcohol Use BPAs

The Alcohol Use Disorder/Risky Alcohol Use BPA will display in the General BPA Section of the navigator when a patient screens positive on the AUDIT or has an alcohol-attributable diagnosis on file. The BPA includes a corresponding SmartSet with orders specific for alcohol use disorder or risky alcohol use depending on the screening result.

BPA Triggers

In the primary care screening workflow, the patient responds to an initial screen consisting of the single item alcohol question. This may be in-person, via MyChart or via a video or telephone rooming process. Depending on the initial screening result, the patient is given the following tool:

- Single item alcohol question response of >=1: AUDIT

A positive score on the AUDIT and/or an alcohol-attributable diagnosis on file will then trigger the BPA as follows:

When AUDIT score is 15 or higher for men, or 13 and higher for women:

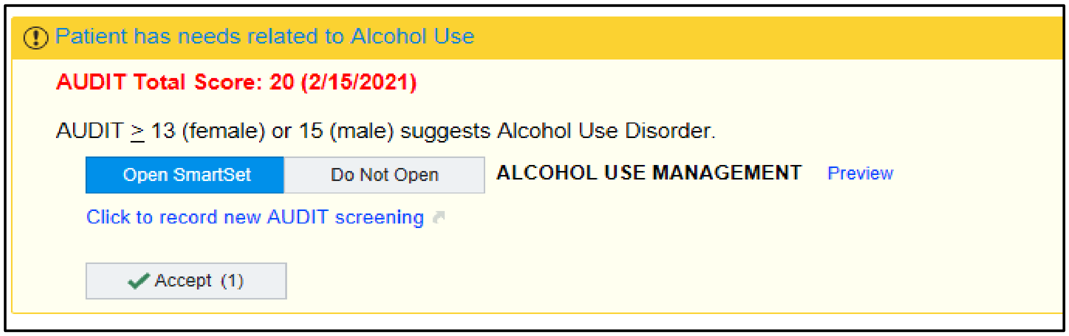

When AUDIT Score is 15 or higher for men, or 13 and higher for women AND a recent alcohol-attributable diagnosis on file:

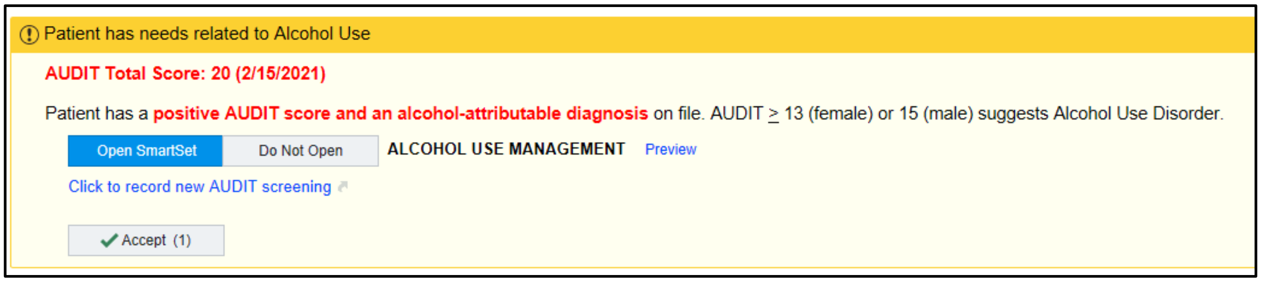

© 2024 Epic Systems Corporation

When a recent alcohol-attributable diagnosis is on file:

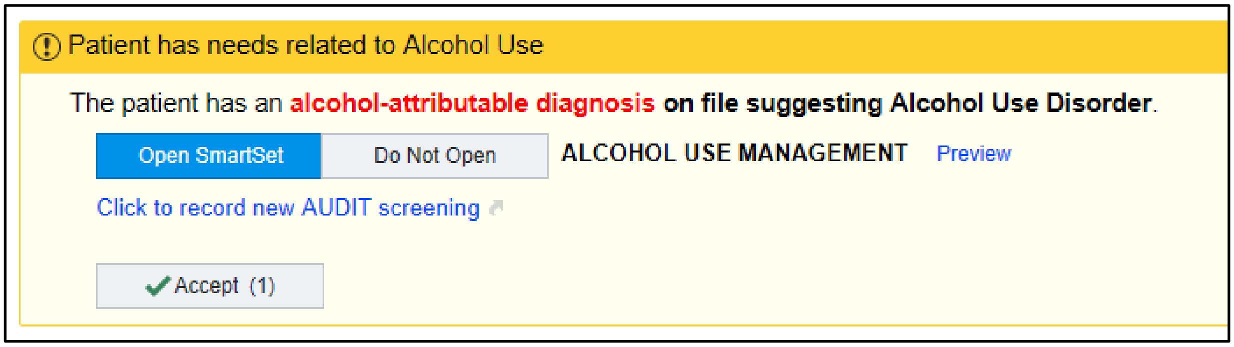

When AUDIT score is between 2 and 15 for men, or between 2 and 13 for women:

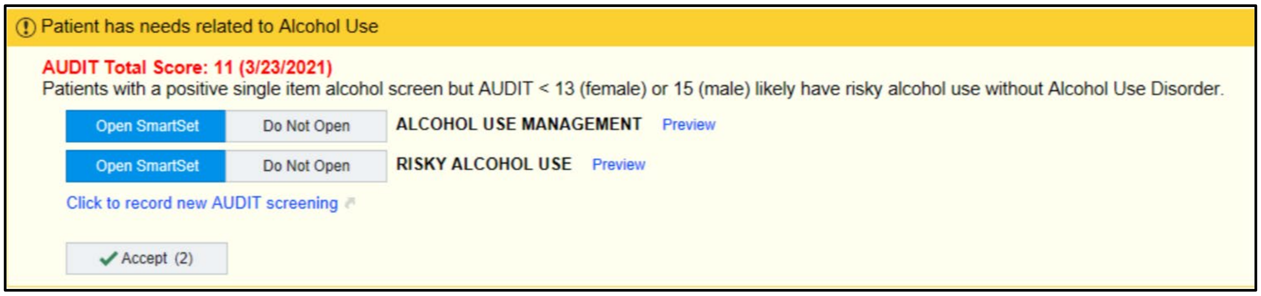

<u>AUDIT Screening Link</u>

Navigate to the General BPA section and click the "Click to record new AUDIT screening" link in the Alcohol Use BPA.

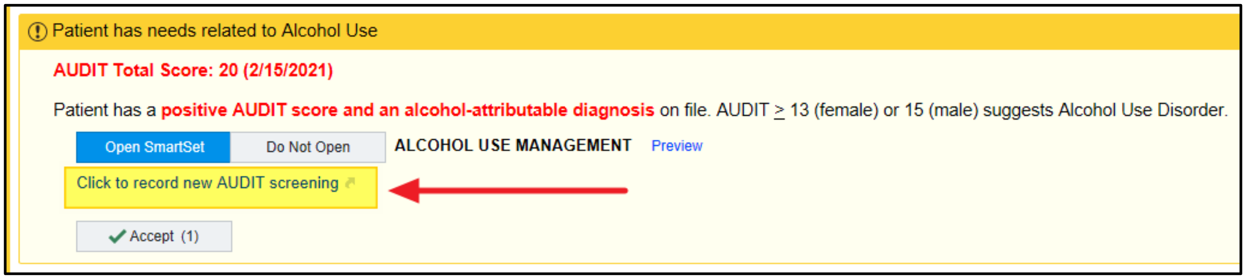

© 2024 Epic Systems Corporation

Single Alcohol Screen Flowsheet will appear:

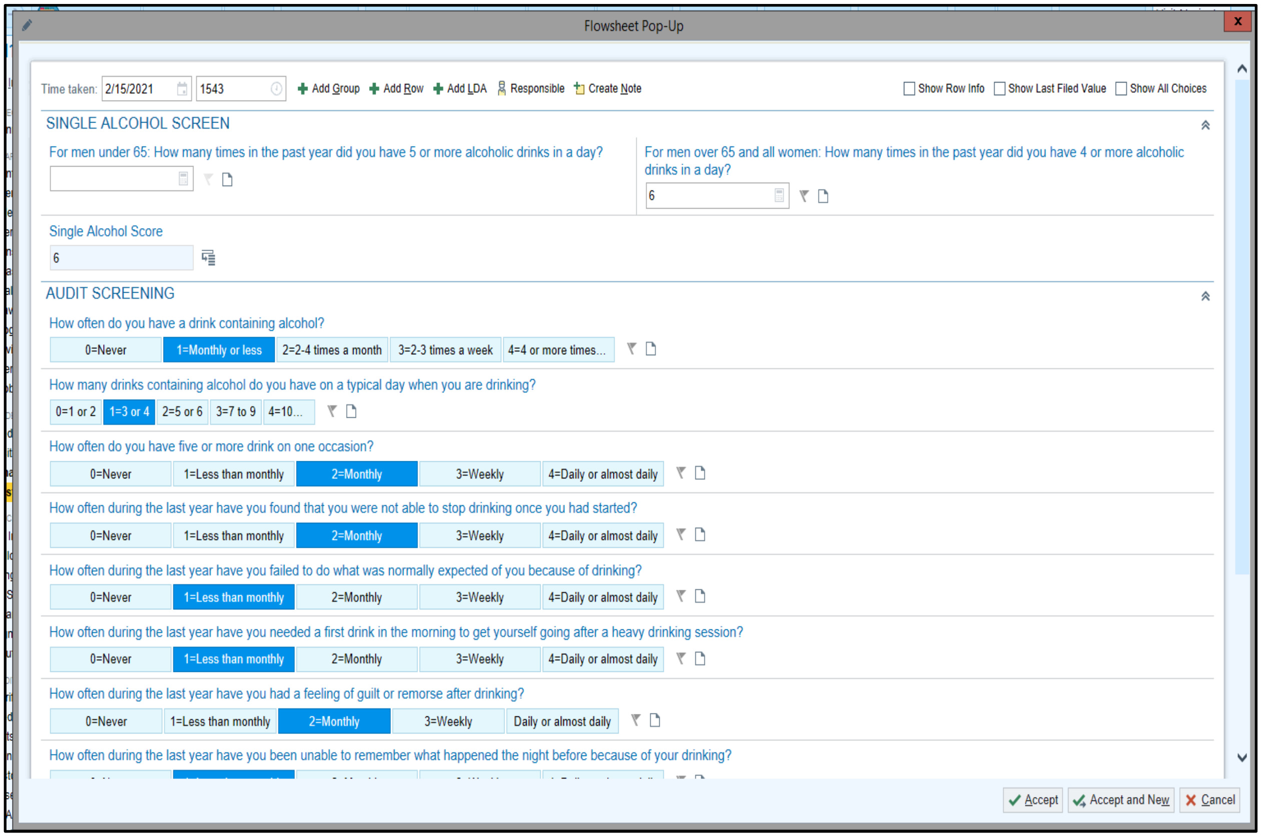

Click "Accept" in the BPA to open the Alcohol Use Management SmartSet.

© 2024 Epic Systems Corporation

Addressing the SmartSets

Each SmartSet includes diagnostic support, medications, labs, referrals, and patient Instructions for the provider to choose from.

RISKY ALCOHOL USE SMARTSET

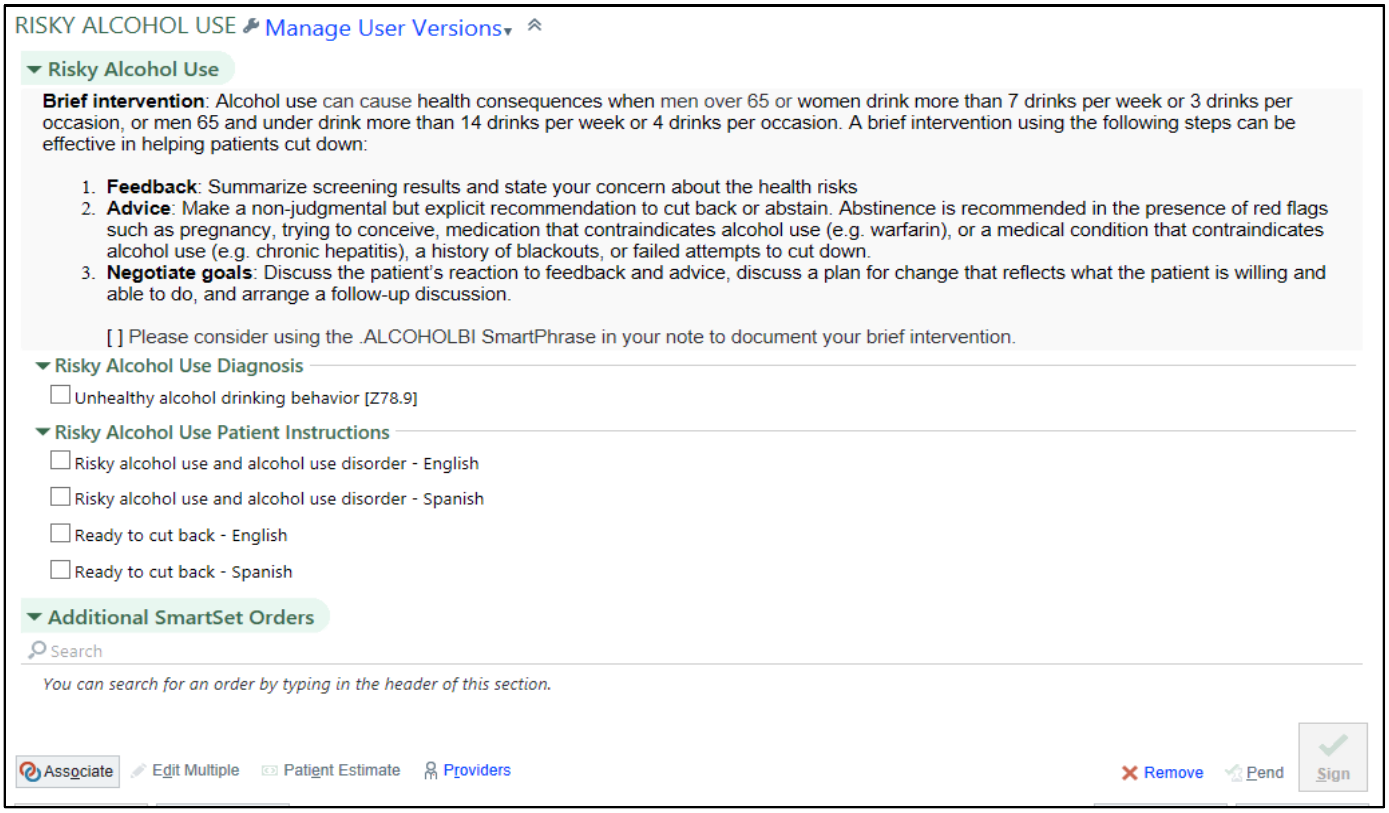

© 2024 Epic Systems Corporation

ALCOHOL USE MANAGEMENT SMARTSET

ALCOHOL USE MANAGEMENT Manage User Versions

From BestPractice

Patient has needs related to Alcohol Use

AUDIT Total Score: 11 (3/23/2021)

Patients with a positive single item alcohol screen but AUDIT < 13 (female) or 15 (male) likely have risky alcohol use without Alcohol Use Disorder.

Click to record new AUDIT assessment

- Click link to record new AUDIT assessment

Alcohol Use Discussion with Patient

Provide Brief Intervention

1. Build rapport:
- "Can I ask you about your alcohol use?"
- "Tell me about your alcohol use."

2. Provide feedback on screening results or recent diagnosis:
- "What do you make of these results (or diagnosis)?"

3. Provide advice. Consider a referral and/or medication.
- "Is it okay if we talk about how alcohol may be impacting you or your health?"
- "Based on my medical experience, I would recommend...What do you think about that?"

Consider suggesting referral and/or medication.

Interactive Handout for Patient Goal Setting - English

Interactive Handout for Patient Goal Setting - Spanish

Alcohol Use Disorder Diagnoses

AUDIT Score Reference

Dx Reference: Diagnostic Checklist

In the past year, has patient experienced the following symptoms:

- Had times when you ended up drinking more, or for longer than you intended?
- More than once, wanted to cut down or stop drinking, or tried to, but couldn't?
- Spent a lot of time drinking, being sick after drinking, or getting over after-effects?
- Experienced craving - a strong need, or urge, to drink?
- Given up or cut back on activities that were important or interesting to you, or gave you pleasure, in order to drink?
- Continued to drink even though it was causing trouble with your family/friends?
- Found that drinking - or being sick from drinking - often interfered with taking care of home/family, caused job troubles, or school problems?
- Had to drink more than you once did to get the effect you want, or found that your usual number drinks had much less effect than before?
- Continued to drink even though it was making you feel depressed or anxious or adding to other health problems, or after having had a memory blackout?
- More than once, gotten into situations while or after drinking that increased your chances of getting hurt (such as driving, swimming, using machinery, walking in a dangerous area or having unsafe sex)?
- Found that when alcohol effects were wearing off, you had withdrawal symptoms, such as trouble sleeping, shakiness, irritability, anxiety, depression, restlessness, nausea or sweating, or sensed things that were not there?

Mild: *2-3 symptoms* ***Moderate:*** *4-5 symptoms* ***Severe:*** *6+ symptoms*

Diagnoses (mild = 2-3 symptoms; moderate = 4+ symptoms)

☐ Mild alcohol use disorder [F10.10]

☐ Moderate or severe alcohol use disorder [F10.20]

Patient's Relevant Lab Results

Relevant labs, if there are results in system (ALT, AST, GFR): *No results found for: ALT, BMCAST, GFR, GFRA*

Rx Reference - AUD Medications

First-line AUD medications (naltrexone, acamprosate)

Medication	Dosing	Side effects and risks	Precautions	Notes
**First-line**				
**Oral Naltrexone**	Start with 25 mg/day and increase to 50 mg/day on day 3 if tolerated.	Nausea, headache, dizziness, elevated transaminases	Will precipitate withdrawal if ongoing opioid use; requires 7 days abstinence from opioids.<br><br>**Contraindicated with AST or ALT > 5 x ULN or decompensated cirrhosis due to impaired metabolism in liver disease. Periodic monitoring of liver function tests (LFTs) is recommended.**	Use as needed (e.g. before a situation in which the patient feels they might drink) can be effective.
**Intramuscular (IM) naltrexone**	380 mg IM every four weeks.	Nausea, fatigue, dizziness, injection site reaction	Will precipitate withdrawal if ongoing opioid use; requires 7 days abstinence from opioids.<br><br>**Contraindicated with AST or ALT > 5 x ULN or decompensated cirrhosis due to impaired metabolism in liver disease. Periodic monitoring of liver function tests (LFTs) is recommended.**	Consider trial of PO formulation first. IM formulation may require prior authorization from insurance.<br><br>Can be used to treat concurrent opioid use disorder.
**Acamprosate**	666 mg by mouth three times daily. CrCl 30-50 ml/min: half dose	Diarrhea, nervousness, fatigue	**Contraindicated with CrCl </= 30 ml/min**	

Medication table adapted from Pace & Samet, Annals of Int Med 2016

Second-line AUD medications (disulfiram, topiramate)

© 2024 Epic Systems Corporation

Medication	Dosing	Side effects and risks	Precautions	Notes
**Second-line**				
**Disulfiram**	Weeks 1-2: 500 mg by mouth daily<br>Thereafter: 250 mg by mouth daily	Drowsiness, metallic taste, headache, peripheral neuropathy, rare fulminant hepatitis	**Patient should not use alcohol 12 hours before and up to three weeks after starting therapy, as causes highly unpleasant aversive reaction.**<br>Contraindicated in severe coronary artery disease; psychosis; pregnancy; or with allergies to rubber, nickel or cobalt.	Most effective when administration is supervised by a professional or significant other, or for an otherwise highly motivated patient.<br>Use as needed (e.g. before a situation in which the patient feels they might drink) can be effective.
**Topiramate**	Start with 25 mg at bedtime, increase by 25-50 mg daily each week, divided into morning and evening.<br>Target does: 300 mg (total daily dose)<br>CrCl < 70 ml/min: reduce dose to 50% of normal dose and titrate more slowly	Cognitive impairement, paresthesias, taste perversion, weight loss, headache, fatigue, dizziness, and depression	**Many possible drug interactions, including but not limited to reduced effectiveness of hormonal birth control; review med list carefully.**<br><br>**Requires baseline and periodic renal function and electrolytes. Titrate slowly with renal impairment.**	**Not FDA approved for treatment of AUD.**

Medication table adapted from Pace & Samet, Annals of Int Med 2016

A. No Liver Disease/No Opioid Use (ALT/AST < 5x Upper Limit of Normal & No Decompensated Liver Disease)

For patients with chronic opioid use, skip to section B below.

First-line Medication

IM naltrexone *is available through the OBAT clinic. Please place referral to OBAT for IM naltrexone.*
Acamprosate: *Consider 2 tablets twice daily in patients less than 60 kg.*

- ☐ naltrexone (DEPADE) 50 mg tablet
- ☐ Referral to GIM: OBAT for IM naltrexone
  Routine 1 visit
- ☐ acamprosate (CAMPRAL) 333 mg tablet

Second-line Medication

Labs

Recommended: *CBC, CMP, RPR, Hep A, Hep B, Hep C, HIV*
If indicated: *urine toxicology screen, urine opioid panel, HCG*

- ☐ Recommended labs (preselected)
- ☐ Urine Toxic Screen and Opioid Panel (if indicated)

Referrals

- ☐ Ambulatory Referral to GIM Integrated BH (GIM PCP ONLY): Social Work Clinician
  Routine 1 visit
- ☐ Ambulatory Referral to GIM Integrated BH (GIM PCP ONLY): Psychologist
  Routine 1 visit
- ☐ Ambulatory Referral to GIM Integrated BH (GIM PCP ONLY): Psychiatric MD/NP
  Routine 1 visit
- ☐ Ambulatory Referral to GIM: OBAT
  Routine 1 visit
- ☐ Ambulatory Referral to Catalyst: Adult Program (18-25 years old)
  Routine 1 visit

Alcohol Use Disorder Patient Instructions - English

Alcohol Use Disorder Patient Instructions - Spanish

B. No Liver Disease/Yes Opioid Use (ALT/AST < 5x Upper Limit of Normal & No Decompensated Liver Disease & Chronic Opioid Use)

First-line Medication

For patients requiring assistance with stopping opioids, a referral to an addiction specialist is recommended.
Acamprosate: *Consider 2 tablets twice daily in patients less than 60 kg.*

- ☐ acamprosate (CAMPRAL) 666 mg tablet

Second-line Medication

Labs

Referrals

Alcohol Use Disorder Patient Instructions - English

© 2024 Epic Systems Corporation

- Alcohol Use Disorder Patient Instructions - Spanish

C. Yes Liver Disease/No Renal Failure (ALT/AST > 5x Upper Limit of Normal OR Decompensated Cirrhosis)

For patients with GFR less than 50 ml/min, skip to section D below.

- First-line Medication

Acamprosate: *Consider 2 tablets twice daily in patients less than 60 kg.*

☐ acamprosate (CAMPRAL) 666 mg tablet

- Second-line Medication (use disulfiram with caution)
- Labs
- Referrals
- Alcohol Use Disorder Patient Instructions - English
- Alcohol Use Disorder Patient Instructions - Spanish

D. Yes Liver Disease/Yes Renal Failure (ALT/AST > 5x Upper Limit of Normal or Decompensated Cirrhosis + GFR < 50)

If GFR is less than 30 ml/min, a referral to an addiction specialist for evaluation is recommended prior to medication initiation.

- First-line Medication

Acamprosate: *Do not use if GFR < 30.*

☐ acamprosate (CAMPRAL) 333 mg tablet

- Second-line Medication (use disulfiram with caution)
- Labs
- Referrals
- Alcohol Use Disorder Patient Instructions - English
- Alcohol Use Disorder Patient Instructions - Spanish

Additional SmartSet Orders

Search

You can search for an order by typing in the header of this section.

Note: Major difference between the A-D sections is medications available for selection.

© 2024 Epic Systems Corporation

SmartPhrases and Tools

The following Behavioral Health SmartPhrases and tools are available for note-writing and reference.

SmartPhrase .AUDIT and .AUDITLASTSCORE for Alcohol Use.
This will pull in the patient's last AUDIT score

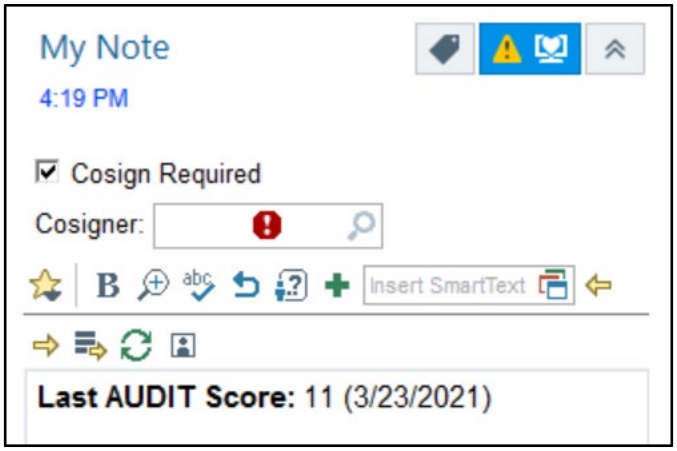

SmartPhrase .ALCOHOLBI for Risky Alcohol Use. (This SmartPhrase is also referenced in the Risky Alcohol Use BPA.)

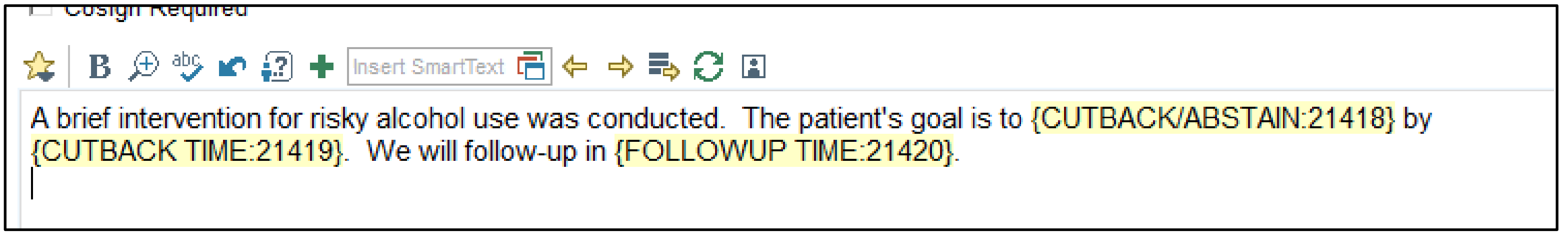

© 2024 Epic Systems Corporation
